# Cold Dry Air as a Therapeutic Measure in Pulmonary Diseases

**Published:** 1875-08

**Authors:** Charles H. Harris

**Affiliations:** Cedar Town, Ga.


### COLD, DRY AIR AS A THERAPEUTIC MEASURE IN THE TREATMENT OF PULMONARY DISEASES.

By CHARLES H. HARRIS, M.D., of Cedar Town, Ga.

It is a difficult matter to introduce a new subject of importance and magnitude to an educated class without being prolix and consuming much time and space. I will, however, make this paper as short as circumstances will admit.

It must have been apparent to every experienced physician that our most approved plans of treating both acute and chronic pulmonary diseases frequently fail. As yet our science has contributed nothing, in a positive sense, to the cure of phthisis, asthma, and many of the forms of chronic bronchitis. The idea of medicine has given place to hygiene and dietetic measures. Extending our observations to the results of treatment in the acute forms of pulmonary disease, we discover that the various plans are attended with uncertainty. Usually they succeed, but sometimes fail, and occasionally there comes along an epidemic which sweeps its victims to the grave in spite of scientific medicine. Now, why, I would ask, will not a disease yield to the same treatment every time, "all things being equal?" The reason is not to be found in the constitution of our patients, nor in the nature of disease. Autopsies reveal the fact that inflammations affect parts now as they ever did, and the best authorities tell us that man and disease are the same to-day they were centuries ago. We are then forced to the conclusion that the cause must be found in the changes which take place in the surroundings of the patient. Among the most important of these are the fluctuations and variations in the air we breathe. Its modifying influence has been noticed by every observing physician. How readily most acute pulmonary diseases yield when our patients are blessed with a cold, dry atmosphere. On the other hand, with what tenacity they cling when the air is damp and warm and heavy. You have noticed this in the mortality attending consumption in the spring and fall seasons. They rarely die in cold, dry weather, but are braced up by it. 'Tis when the "sap rises" and the "leaves fall" that this long caravan takes up its march "across the river." The people even know that a summer cold is worse than a winter one. The reason is very plain: the warm air of summer relaxes and debilitates the capillary system, and readily admits of congestions and inflammations. The cold air of winter is tonic, and it is an excellent local remedy for an inflamed mucous membrane. I venture the assertion that an epidemic of pneumonia never had its origin in a cold, dry atmosphere. On the contrary, there are few epidemics that will not die out in very cold, clear weather. Hence we see the importance of a cold, dry air, and it is with a view of directing the attention of physicians to the fact, and applying it to the treatment of disease, that this paper is written.

It is with diffidence that I announce to the profession that we can supply our patients with an abundance of pure, cold air at any time. The apparatus I use is simple in structure, cheap and may be put up by any physician in a few moments. It consists of a gutta percha tube eight or ten yards in length, (the longer the better) a large cork stopper and an ordinary water bucket. A lead tube would answer better, as it conducts heat well and is not affected by water or air. The tube has a mouthpiece at one end and a vulcanized terminus at the other, both open. Near the bottom of the bucket, through a stave, I bore a hole to fit the cork. I also bore a hole through the cork to fit the vulcanized terminus. Having inserted the terminus through the cork, I then stop the hole in the bucket, from the inside, with the cork. This allows a portion of the terminus to project outside of the bucket. I then carefully coil the tube on itself up the inside wall of the bucket, after the manner of a still-worm, leaving a foot of the tube, with the mouthpiece, hanging from the top. The bucket is then filled with ice water or any refrigerating mixture. After waiting a few minutes for the tube to get thoroughly cold, let the patient take the mouthpiece between his lips. Tell him to inhale through the tube and exhale through the nose.

The changes which take place in the air as it passes through this long, cold tube are easily determined. Setting aside those of a chemical nature, we will examine them from a common sense standpoint. Those who have noticed the "sweating" of a tumbler of ice water will readily see that the air in passing its circuitous route over a cold surface will part from some of its moisture and a "sweating" will take place inside of the tube. If the tube is long enough and the water cold enough, this sweating will amount to a dripping, which will trickle along down the tube and finally drop out from the lower terminus. In this sweating process the air evidently becomes dry, and in all probability loses many of its impurities. That the air grows cold in proportion to the length of the tube is plain to any one who tries it. If ice is not at hand, and you use spring water, the air is even then perceptibly colder in the tube than outside. Patients will tell you this, and though water may be dripping from the lower end of the tube, they will notice that the air is dry. Cold reduces the volume of everything except water in freezing, and it is safe to conclude that in passing through this cold tube the volume of the air is greatly reduced. Vapors expand and contract more in proportion to their volume under thermal changes than fluids and solids. This reduction of volume I think an important item, as the blood can be supplied with oxygen with less labor to the respiratory organs. The air grows cold in its transit and becomes an invaluable local remedy to the inflamed bronchial mucous membrane.

To sum up, we have a cold, dry air, robbed, in all probability, of many of its impurities, and, by reason of reduced volume, rich in oxygen. Would not a patient with any pulmonary disease thank you for such air? Aye, more, would not any enfeebled, debilitated system rally under its use? Would it not be bracing to the healthy and invigorating to the invalid? I would sooner risk it than medicated vapors and atomized liquids. I think it much safer than plunging the aspirator in a network of vital parts. Does it not promise all the benefits that may be derived from a change of climate? If one can get pure, bracing air at home, is it not better in the bosom of the family than a stranger in a strange land? As to economy, the appliance is cheap, may be put up by any one, and is within the reach of every doctor. The subject I think one of importance and magnitude, and will commend itself to a thinking mind.

Without writing more, I commit it to you who have hospitals and material at your disposal for future investigation, with the request that you give it an earnest and diligent study. Should you have a patient prostrated from any chronic or incurable attack, let him use the "pure air condensed" for two hours before each meal. Try it in cases of phthisis and bronchitis, and every form of obscure pulmonary disease. Try it in scrofula, croup, asthma, *et id omne genus*.

